# Embryonic Stem Cells Derived Kidney Organoids as Faithful Models to Target Programmed Nephrogenesis

**DOI:** 10.1038/s41598-018-34995-3

**Published:** 2018-11-09

**Authors:** Zenglai Tan, Jingdong Shan, Aleksandra Rak-Raszewska, Seppo J. Vainio

**Affiliations:** Biocenter Oulu, Infotech Oulu, Center for Cell Matrix Research, Faculty of Biochemistry and Molecular Medicine, University of Oulu, Aapistie 5A, 90220 Oulu, Finland

## Abstract

The kidney is a complex organ that is comprised of thousands of nephrons developing through reciprocal inductive interactions between metanephric mesenchyme (MM) and ureteric bud (UB). The MM undergoes mesenchymal to epithelial transition (MET) in response to the signaling from the UB. The secreted protein Wnt4, one of the Wnt family members, is critical for nephrogenesis as mouse Wnt4^−/−^ mutants fail to form pretubular aggregates (PTA) and therefore lack functional nephrons. Here, we generated mouse embryonic stem cell (mESC) line lacking *Wnt4* by applying the clustered regularly interspaced short palindromic repeats (CRISPR)/CRISPR-associated systems 9 (Cas9). We describe here, differentiation of the wild type and *Wnt4* knockout mESCs into kidney progenitors, and such cells induced to undergo nephrogenesis by the mouse E11.5 UB mediated induction. The wild type three-dimensional (3D) self-organized organoids depict appropriately segmented nephron structures, while the *Wnt4*-deficient organoids fail to undergo the MET, as is the case in the phenotype of the *Wnt4* knockout mouse model *in vivo*. In summary, we have established a platform that combine CRISPR/Cas9 and kidney organoid technologies to model kidney development *in vitro* and confirmed that mutant organoids are able to present similar actions as in the *in vivo* studies.

## Introduction

The mammalian metanephric kidney develop from the interaction between the UB and MM cell populations, including the *Six2*+ *Cited1*+ nephron progenitor cells (NPCs) and *Foxd1*+stromal precursor cells^1–5^. The UB undergoes branching morphogenesis to form the tree-like collecting system^4^. The tips of the UB signal to the MM to maintain undifferentiated NPCs (*Six2*+*Cited1*+) and induce the differentiation in a subset of NPCs (*Six2*+*Cited1*−)^5^. The latter NPCs begin to aggregate to form the PTAs and undergo MET and become polarized, and form renal vesicles (RVs) with a lumen. RVs will sequentially transition to Comma-shaped bodies, and S-shaped bodies, eventually forming segmented nephrons, including glomeruli and adjacent proximal tubules and distal tubules^6^.

The *Wnt4* gene encodes a signaling glycoprotein and it is expressed in multiple organs such as the embryonic metanephric kidney, the adrenal gland, the bipotential gonad, and the mammary and pituitary glands, and it plays an important role in organogenesis^7–10^. A homozygous missense mutation in the human *WNT4* gene causes SERKAL (SEx Reversion, Kidneys, Adrenal and Lung dysgenesis) syndrome, which leads to fetal lethality^11^. Conventional *Wnt4* knockout mouse embryos manifest several deficiencies; the kidney development is impaired at an early stage and the MET fails^10^. *Wnt4* is expressed at the comma and S-shape stages of nephrogenesis; complete inactivation of *Wnt4* in mice leads to early postnatal death, almost certainly due to the lack of kidney function^10^. *Wnt4* signaling also controls the differentiation of the stromal cells in the embryonic kidney^12^. All these data shows that *Wnt4* plays an important role during kidney development *in vivo*. However, we fail to know if *Wnt4* provides such functions in developing kidney organoids *in vitro*.

Protocols to generate human pluripotent stem cells (hPSCs)-derived renal organoids to model human kidney development and diseases have been recently published^13–17^. Using appropriate chemical compounds or growth factors, developmental signaling pathways can be triggered to promote PSCs differentiation into nephron progenitors. The hPSCs-derived nephron progenitors can also undergo MET and generate mature nephrons and collecting duct structure*s*^13–17^. The 3D kidney culture technology, allow human and mouse PSCs to exhibit their remarkable self-organizing properties depicted by appropriately segmented structures of nephrons^14,16^.

The genome engineering technique, the CRISPR/Cas9 gene editing, provides an unprecedented opportunity for studying kidney disease and development with hPSCs *ex vivo*. These techniques provide new resources for modelling and studying human kidney development and disease. The induction of renal lineage has been conducted using mESCs^18–24^ and these mESCs-derived nephron progenitor cells can be induced to nephron structures by spinal cord^17^. These findings show that mESCs have important potential for modelling the development as well as regeneration.

Here, we report a novel setting to be able to combine CRISPR/Cas9 with kidney organoid technologies to model kidney development. By using the CRISPR/Cas9 technique, we generated the *Wnt4* deficient mESCs. We programmed differentiation of wild type and mutant mESCs into kidney progenitors and through the interaction with UB were able to induce nephrogenesis and generate kidney organoids *ex vivo*. We demonstrate that the *Wnt4* CRISPR-knock out cells, generate kidney organoids which fail to advance the MET and lead to failure in nephrogenesis. Taken together, these results depict an innovative platform for mouse kidney development modelling and regenerative medicine application for detailed molecular genetic studies.

## Results

### Generation of *Wnt4* deficient mESCs with double nicking by RNA-guided CRIPSR/Cas9

**Figure 1:**
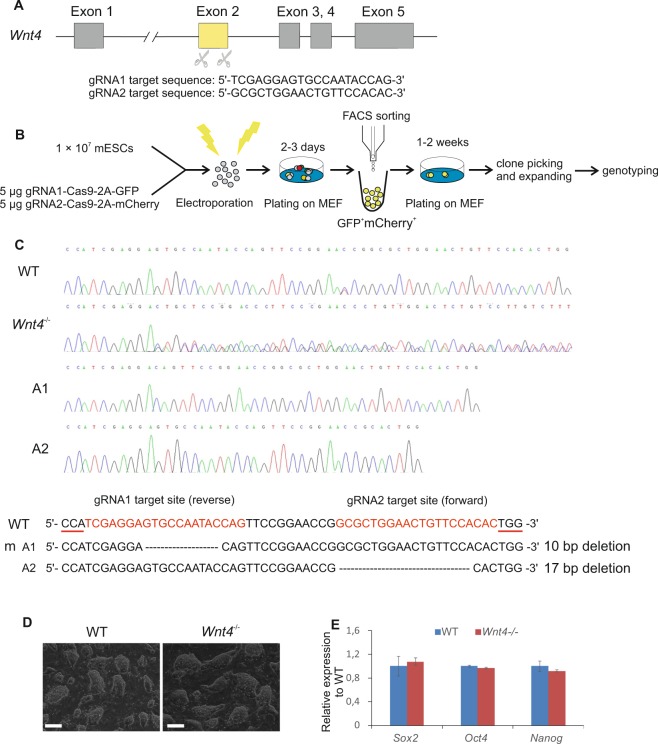
Generation and characterization of *Wnt4* knockout mESCs. (**A**) Schematic diagram of the location and sequences of the two sgRNAs designed to target the exon 2 of the *Wnt4* gene. (**B**) Schematic of the double nicking by RNA-Guided CRISPR/Cas9 knockout of *Wnt4* in mESCs. MEF: mouse embryonic fibroblast. (**C**) Chromatogram of the representative wild type and CRIPSR/Cas9 *Wnt4* mutant clone. Interpretation shows separated alleles (A1 and A2) aligned against the wild type sequence. The red line represent the PAM sequence while the dotted lines indicate deletions. (**D**) Representative bright field images of undifferentiated wild type mESCs, and *Wnt4* knockout mESCs colonies. The colonies look alike and cells do not present any differences in formation of the colonies. Scale bars: 200μm. (**E**) qRT–PCR results show the expression level of the stem cell markers (*Sox2*, *Oct4*, *Nanog*) – no significant differences between wild type mESCs and *Wnt4* knockout mESCs can be observed.

The mouse *Wnt4* gene consists of five exons; previously reported conventional *Wnt4* knockout mouse model, generated a probable null allele by replacing the whole exon 3 with a *neo* selection cassette^10^. To analyze the role of *Wnt4* during kidney organoid development *in vitro*, we generated a *Wnt4* deficient mESC line using the CRISPR/Cas9 genome editing technology. We used a pair of small guided RNAs (sgRNAs) guiding paired Cas9 nickases to knockout genes in mESCs, which have been shown to reduce the off-target activity and facilitate gene knockout efficiency in cell lines^25^. We designed the sgRNAs to target *Wnt4* exon 2 (Fig. 1A), and constructs encoding GFP or mCherry-tagged Cas9 and sgRNAs were electroporated into the wild type mESCs. GFP and mCherry co-expressing cells were FACS sorted and positive clones were picked and expanded (Fig. 1B). Sanger-sequencing results revealed the knockout mESC line with one allele 10 bp and another allele 17 bp deletion in the *Wnt4* exon 2 (Fig. 1C).

We observed that the *Wnt4*^−/−^ mESCs colonies were indistinguishable in size and shape from unmodified mESCs (Fig. 1D), and presented similar expression level of mESC markers such as *Sox2*, *Oct4* and *Nanog* (Fig. 1E and Supplementary S1A), indicating that the *Wnt4*^−/−^ cells maintained pluripotency and self-renewal properties.

### Induction of intermediate mesoderm differentiation in the mouse ES cells

**Figure 2:**
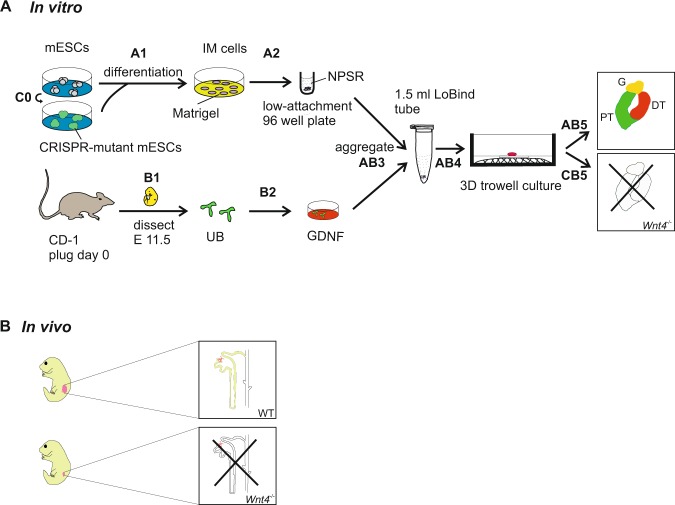
Schematic representation of *Wnt4* role during kidney development *in vivo* and *in vitro*. (**A**) *In vitro* model of Wnt4 role during kidney organoids development. (Step A1) Direct differentiation of mESCs into intermediate mesoderm (IM) cells in monolayer cultures. (Step A2) IM cells differentiation into nephron progenitors (“priming” of the pellet). (Step B1) Dissection of the UB from CD-1 mouse embryos at E11.5. (Step B2) Incubation of the UB with hGDNF for 30 min at 37 °C. (Step AB3) Aggregation of the mESC derived-nephron progenitors and hGDNF treated UB as a 3D pellet and incubation in 1.5 ml Eppendorf LoBind tube overnight. (Step AB4) Transfer of the 3D pellet to Trowel culture in DMEM basic medium supplemented with 10% FBS. (Step AB5) Maturation of the kidney organoids - giving rise to nephron structures. (Step C0) CRISPR/Cas9 knockout of *Wnt4* in the mESCs. (Step CB5) Failure of kidney development in *Wnt4* knockout organoids. (**B**) *In vivo* model of Wnt4 function during kidney development.

**Figure 3:**
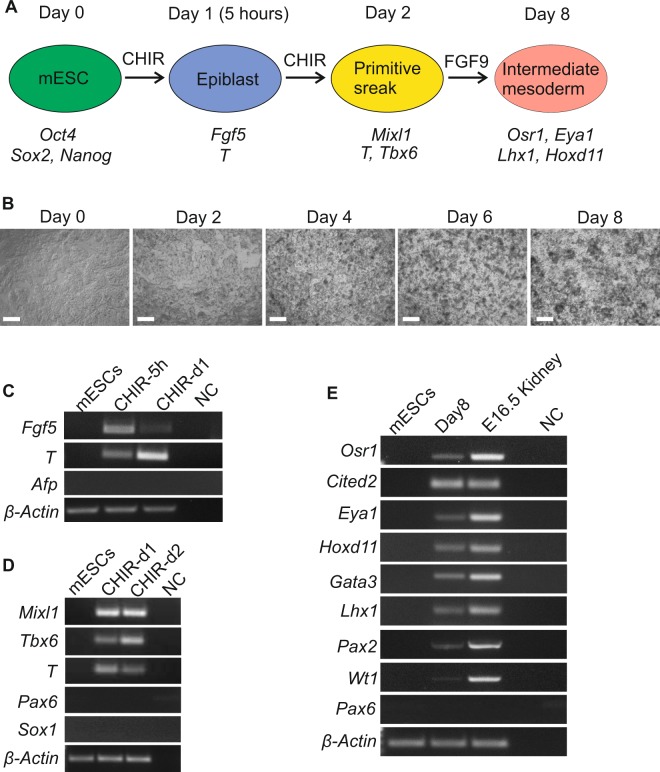
Direct differentiation of mESCs to IM. (**A**) Schematic protocol of induction of IM from mESCs. (**B**) Phase contrast images of mESCs in monolayer (2D) cultures during differentiation into IM. Consecutive days are shown, with day 0 indicating the time point immediately before CHIR99021 treatment. Scale bars, 200 µm. (**C**) RT-PCR of mESCs differentiation presenting gene expression changes during CHIR treatment, after 5 hours and 1 day; cells expressed epiblast markers *Fgf5* and *T* but no extraembryonic endoderm marker *Afp*. (**D**) RT–PCR presenting gene expression changes during further CHIR differentiation of mESCs; day 1 and 2 showing the expression of primitive streak markers (*Mixl1*, *T*, *Tbx6*) but no ectodermal markers (Pax6 and Sox1). (**E**) RT–PCR at day 8 of differentiation showing the expression of markers of IM (*Osr1*, *Pax2*, *Lhx1*, *Gata3*, *Wt1*, *Eya1*, *Cited2*, *Hoxd11*) while ectodermal marker Pax6 was not detected. E16.5 Kidney cDNA was used as a positive control. NC, negative control with no DNA template. (**C–E**) Full-length gels are presented in Supplementary Information.

In order to use the CRISPR *Wnt4* knockout mESCs to model kidney development *in vitro* we have optimized protocol to generate kidney organoids from wild type mESCs. mESCs were isolated from mouse blastocysts at E3.5^26,27^. Activation of LIF-Stat3 or Wnt/β-catenin signaling promotes mESCs self-renewal^28–30^ and CHIR99021, an inhibitor of the GSK-3, induces non-neural differentiation^28^. We have therefore treated the mESCs with the 5 h pulse of CHIR99021 in monolayer cultures. Cells presented expression of the epiblast markers such as *Fgf5* and *T* (*Brachyury*) but not the extraembryonic endoderm marker *Afp* (Fig. 2A, step A1 and Fig. 3A,C), suggesting direct epiblast differentiation. At 24 hours, *Fgf5* was downregulated while the *T* marker was upregulated depicting early primitive streak differentiation (Fig. 2A, step A1 and Fig. 3A,C).

The canonical Wnt signaling pathway induces primitive streak development in mESCs and hESCs^31,32^. The intermediate mesoderm (IM) arises from the primitive streak. *Fibroblast growth factor 9* (*Fgf9*) is expressed in IM^33^ and FGF9 signaling supports MM differentiation *in vitro*^34^. Previous studies demonstrated that differentiation of IM from hPSCs requires CHIR99021-to-FGF9^13,14^, we therefore analyzed whether mESCs-derived epiblast cells reacted similarly to the Wnt and FGF signaling. We treated these cells with CHIR99021 for 48 h and found expression of primitive streak markers such as *Mixl1*, *T* and *Tbx6* (Fig. 2, step A1 and Fig. 3A,D), implicating early mesendoderm differentiation. Indeed, FGF signaling was required for generation of the IM cells. In the presence of FGF9 and heparin, these cells advanced their differentiation and their growth dynamics changed from a monolayer-type towards cellular clusters (Fig. 3B). The cells isolated at day 8 of differentiation expressed *Osr1*, *Pax2*, *Lhx1*, *Wt1*, *Ctied2*, *Eya1*, *Hoxd11* and *Gata3* genes (Fig. 3E). These genes are typically expressed in the cells of IM associated with kidney lineages^19^. In summary, we have established a novel protocol to induce the mESCs towards the IM stage in a monolayer culture setting with CHIR99021-FGF9/heparin supplementation (Fig. 3A).

### Generation of kidney organoids from mESCs-derived nephron progenitors with embryonic UB

**Figure 4:**
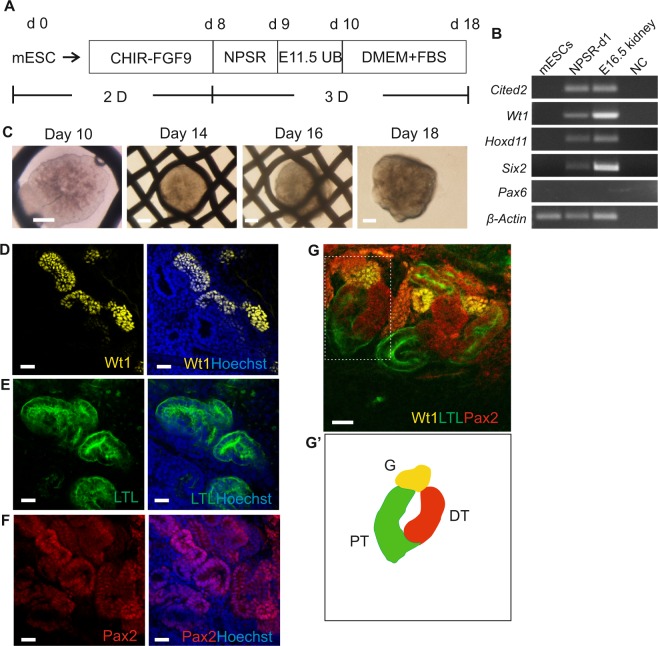
Generation of kidney organoids through aggregation of mESC-derived nephron progenitor cells and embryonic UB. (**A**) Schematic of the differentiation protocol of mESCs into kidney organoids. (**B**) Electrophoresis gel of RT-PCR products presenting kidney lineage cells expressing nephron progenitor markers *Cited2*, *Wt1*, *Hoxd11*, and *Six2* after incubation in NPSR medium overnight. (**C**) Global bright field images of kidney organoids in Trowel culture. Scale bars: 500 μm. (**D–F**) Whole-mount immunofluorescence analyses of the organoids showing nephron progenitor markers: (**D**) glomeruli marker - Wt1, (**E**) proximal tubule marker - LTL, (**F**) nephron marker - Pax2. Scale bars: 20μm. (**G**) Confocal image showing three compartments of segmented nephron, including the distal tubule (DT, Pax2 + LTL−), proximal tubule (PT, LTL+) and the glomerulus (G, Wt1+). Dotted box shows single nephron that was used to generate a schematic diagram in (G’). Scale bars: 20 μm.

Nephron Progenitor Cells (NPCs, Six2+ cells) purified from mouse embryonic kidney present long-term self-renewal properties when cultured in the medium enabling NPC self-renewal (NPSR)^35^. The NPSR medium mimics the *in vivo* nephron progenitor niche by delivering important small molecules and necessary growth factors to maintain undifferentiated progenitor cell proliferation and self-renewal^35^. To test, whether NPSR can indeed induce the NPCs from the mESCs-derived IM cells, we harvested these cells at day 8 of differentiation and cultured them as 3D pellets in the NPSR medium overnight (Fig. 2, step A2 and Supplementary Fig. S2A). This process enhanced expression of the NPC markers, such as *Cited2, Wt1, Hoxd11, and Six2* (Fig. 4A,B). Incubation of the mESCs-derived IM cells in the NPSR medium overnight was a necessary step in priming these IM cells for renal differentiation. If this step was omitted, the nephrogenesis in the IM cells failed to be induced by the UB (Supplementary Fig. S2C).

We reported recently that the UB separated from mouse embryos (E11.5) induces MET and nephrogenesis in an intact and dissociated to single cells and re-aggregated E11.5 MM^36^. Hence, we have used the mouse E11.5 UB tissue as a potent nephrogenesis inducer. We aggregated “primed” 3D pellets with the UB and cultured in 3D Trowell culture system. This led to successful nephrogenesis induction (Fig. 2, steps B1-AB5 and Fig. 4C) depicted by positive staining for glomerulus marker: Wilms tumor 1 (Wt1+, yellow; Fig. 4D); proximal tubule marker: *Lotus tetragonolobus* lectin (LTL+, green; Fig. 4E); and distal tubule marker: (Pax2+ LTL−, red; Fig. 4F), suggesting proper differentiation of cultured organoids to major segments of the nephrons. We also found numerous Wt1+ glomeruli adjacent to the LTL+ proximal tubules, and LTL+ proximal tubules connected with Pax2+ LTL− distal tubules (Fig. 4G, G’).

To verify, that the kidney organoid structures were generated via the interaction between the UB and the mESCs-derived kidney cells, but not by contaminated UB tip cells with the primary MM cells, we cultured the E11.5 UB tissue in isolation in the 3D culture. The UB cells underwent apoptosis already at the second day of culture and died at day 3 (Supplementary Fig. S2B). In addition, there is lack of nephron structures formation when “un-primed” IM cells were aggregated with UB or the IM cells “primed” in NPSR medium were transferred to 3D culture without integration with the UB (Supplementary Fig. S2C,D). These data suggest that the mESCs-derived IM cells can differentiate into nephron progenitors via a “priming step”. Such cells are also competent to undergo the MET and generate 3D kidney organoid when induced with the embryonic UB.

### *Wnt4* regulates nephrogenesis in kidney organoids

Wnt-signaling play multiple roles in different tissues during development. It regulates the pattern formation, cell fate choices, cell renewal, proliferation and migration^28,37–40^. *Wnt4* is important for kidney development; it is the mesenchymal signal for epithelial transformation of MM in the developing kidney^10^ and is expressed in the kidney mesenchyme and its derivatives, namely the nephrons^41–44^. Mouse mutants with loss of *Wnt4* fail to form PTAs and therefore lack functional nephrons^10^.

**Figure 5:**
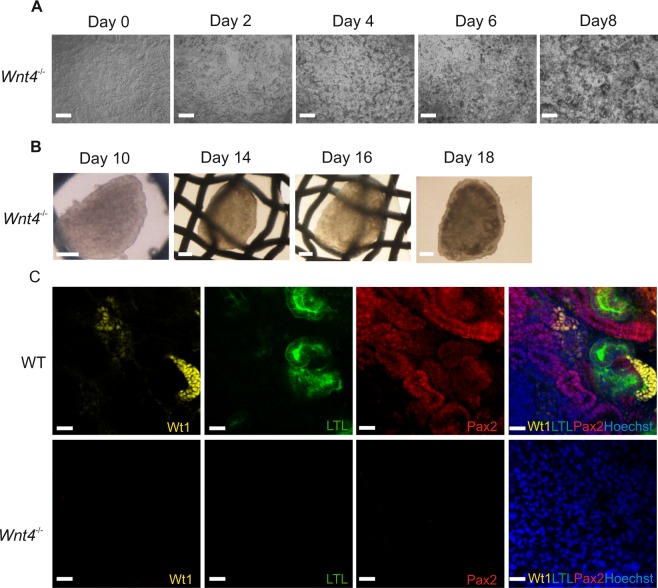
Lack of mesenchyme to epithelial transition (MET) in *Wnt4* CRISPR-mutant kidney organoids. (**A**) Phase contrast images of Wnt4 knockout mESCs in monolayer (2D) cultures during differentiation into IM. Consecutive days are shown. Scale bars: 200 µm. (**B**) Global bright field images of the Wnt4 knockout kidney organoids in Trowel culture. Scale bars: 500μm. (**C**) Immunological characterization of structures within wild type and Wnt4 knockout kidney organoids. Scale bars: 20 μm.

To investigate whether *Wnt4* would play the same role during kidney organoid development, we generated the *Wnt4*^−/−^ organoids with the CRISPR/Cas9 knockout *Wnt4* mESC line. The differentiation conditions of *Wnt4*^−/−^ organoids were the same as the wild type mESCs organoids (Fig. 2A). There is no visible morphology difference between wild type and *Wnt4*^−/−^ mESCs when differentiated to IM population (Fig. 5A and Supplementary S2B); 3D pellets show no distinction before and after priming step (Supplementary S2A and S3D). In addition, *Wnt4*^−/−^ mESCs differentiated to kidney lineage present expression of the same markers as the wild type cells during all differentiation stages (Supplementary S3A–C, E,F). These data show that *Wnt4*^−/−^ mESCs were able to differentiate into kidney lineage.

We used these *Wnt4*^−/−^ kidney lineage cells to aggregate with the wild type UB to make the organoids (Fig. 2A, step CB5). In contrast to the wild type organoids (Fig. 4C), the 3D morphology of the *Wnt4*^−/−^ organoids appeared to be flatter (Fig. 5B). These organoids failed to undergo nephrogenesis and did not generated any kidney structures, as depicted on Fig. 5C with immunostaining for nephron specific markers (Fig. 5C and Supplementary Fig. S4A). The, *Wnt4*^−/−^ organoid failed to undergo the MET and subsequently kidney development failed, as is the case in the *in vivo* model (Fig. 2A,B and Supplementary Fig. S4B). In conclusion, *Wnt4* signal is crucial to regulate epithelial transformation of nephron progenitors in the developing kidney organoids. Moreover, organoids are a good tool to study and model development.

## Discussion

Recent advances in genome editing and stem cell-derived kidney organoid technologies provide the possibility to perform sophisticated genetic studies in PSC-derived kidney lineage. Several groups reported using gene-editing settings to study kidney development and disease in the organoids. Recapitulated features of glomerular and tubular diseases by transiently transfecting undifferentiated hPSCs with plasmids expressing wild type Cas9 and sgRNAs targeting disease-relevant genes *PODXL* (*podocalyxin like*), polycystic kidney disease (PKD) genes PKD1 and PKD2^45,46^ and TALEN knockout *PAX2* to study UB development *in vitro*^16,47^. Here, we report for the first time using the double nicking by RNA-guided CRISPR/Cas9 technique to knockout of *Wnt4* in the mESCs and study kidney development *in vitro*.

Mouse ESC lines possess the ability to differentiate into a variety of cell types, and therefore are a source of cells for functional studies^48,49^. Usually, mESCs differentiation starts with embryoid body (EB) formation as a way of following normal developmental events that take place in the embryo^50^. However, here we have developed a 2D monolayer culture method to successfully differentiate mESCs into epiblast. We further differentiated the epiblast stage cells into IM cell population using CHIR99021-FGF9/heparin treatment. Our studies revealed that using Wnt and Fgf signaling we could efficiently differentiate mESCs into kidney precursor cells in 2D cultures. With previously reported high efficiency of maintaining nephron progenitor’s stemness in NPSR medium^35^, we managed to direct the differentiation of IM into nephron progenitors in a 3D pellet in this medium. This incubation - “priming” step, appeared to be crucial in derivation of NPC from IM that would be competent to undergo nephrogenesis. Our method illustrates that the nephron progenitors derived from mESCs gain the potential to interact with the functional UB and are competent to advance the nephrogenesis.

Human *WNT4* mutation causes kidneys dysgenesis syndrome and mouse knockout of *Wnt4* leads to a failure in kidney development (kidney agenesis). Our studies of *Wnt4* knockout in mESCs illustrate that combining of CRISPR/Cas9 genome editing technology to determine the function of specific genes in different mouse cell types, with 3D generation of organoids leads to similar effects as an *in vivo* study. Moreover, we believe that the mESC *Wnt4*^−/−-^derived NPCs behave similarly to MM. These cells express many markers of competent MM before combining them with freshly isolated UB (Supplementary Fig. S3E), however, after 8 days of culture as an organoid, the Wt1 and Pax2 are lost (Fig. 5) and there are no nephrons present. Normally, MM stays competent for induction signal from the UB for 24–36 h^51^, and lack of it leads to apoptosis of these cells. Also UB undergoes apoptosis if no signals reach it from the MM^52^ (Supplementary Fig. S2B). Given that, in these mutant organoids the Wnt9b from the UB has no cells to act upon (all being *Wnt4*^−/−^)^10^, it is no surprising that the nephrogenesis fails and all MM markers are lost.

The CRISPR/Cas9 technology has enabled efficient creation of various disease models, in our work and of others^45,53^ proving that generation of renal organoids from gene-modified PSCs provide an excellent tool and endless possibilities to model kidney development and disease. These are important breakthroughs, which will promote the development of regenerative medicine.

In summary, we showed here wild type mESCs-derived nephron progenitors aggregated with primary UB formed kidney organoids with full nephron structures. While genome-modified nephron progenitors (*Wnt4*^−*/*−^) aggregated with UB formed mutant organoids with failed kidney development, which functionally recapitulate kidney development phenotypes *in vivo*. The described methodologies (Fig. 2A) are broadly relevant for functional studies of factors involved in development and their potential in regenerative medicine. In the long term, this system may provide a useful setting that will benefit personalized medicine and gene therapy.

## Materials and Methods

The animal care and experimental procedures in this study were in accordance with Finnish national legislation on the use of laboratory animals, the European Convention for the protection of vertebrate animal used for experimental and other scientific purposes (ETS 123), and the EU Directive 86/609/EEC. The animal experimentation was also authorized by the Finnish National Animal Experiment Board (ELLA) as being compliant with the EU guidelines for animal research and welfare.

### Mouse ESCs culture

All experiments used the wild type mouse embryonic stem cells derived from Taconic’s W4/129S6 inbred mouse strain. Undifferentiated wild type and the CRISPR/Cas9 knockout mESCs were maintained on the mouse embryonic fibroblasts (MEFs) as a feeder layer with mESCs medium as previously reported^54^.

### CRISPR/Cas9 genome editing

Cas9 nickase was used for editing the second exon of the *Wnt4* gene following the protocol of the Zhang Feng’s lab, MIT (https://www.addgene.org/crispr/zhang/). pSpCas9n (BB)-2A-GFP (AddGene: PX461) was modified by replacing 2A-GFP with 2A-mCherry. Paired oligoes corresponding to *Wnt4* gRNA1 (5′-TCGAGGAGTGCCAATACCAG-3′) were cloned into pSpCas9n (BB)-2A-GFP vector. Paired oligoes corresponding to *Wnt4* gRNA2 (5′-GCGCTGGAACTGTTCCACAC-3′) were cloned into pSpCas9n (BB)-2A-mCherry vector. Paired GFP and mCherry constructs were co-electroporated into mESCs. GFP and mCherry double positive cells were isolated by flow cytometry sorting (FACS) two days after electroporation, and immediately plated onto the 10 cm MEF coated plate. One to two weeks later, there were colonies growing in the culture plate. Using 100 μl pipette tips we picked up the colonies and placed them individually in the 96 well culture plate filled with trypsin. After dissociation into single cells, the colonies were transferred onto MEF-coated 24-well plates and then expanded in 10 cm plates separately. Further, the genotyping by PCR/TA-cloning and chromatogram sequencing were used to analyze the mutations and select the positive clones for analysis and differentiation experiments. The primers (5′–3′) used for genotyping: *Wnt4* forward: GTATCACATCCAACCACTG, reverse: AGAAGCCTGATGCCAAGGGA.

### Cell differentiation

Mouse ESCs were cultured in Matrigel-coated 6 cm culture dishes, in mESCs medium until reaching 70–90% confluency. mESCs were passaged on Matrigel-coated 6 cm plates at 30,000 cells/cm^2^. Next day, cells reached 80–90% of confluency and were treated with 8 μM CHIR99021 in APEL basal medium (STEMCELL Technologies) for 4 days, followed by FGF9 (200 ng/ml) and heparin (1 μg/ml) treatment for another 4 days; medium was changed every other day. Following the differentiation, there were some floating cells; these were apoptotic cells and were removed during changing medium.

### 3D kidney organoids formation

At day 8 of differentiation, cells were collected and dissociated into single cell suspension using TrypLE select (Life Technologies). Cells (3 × 10^5^) were centrifuged at 1000 rpm for 4 min to form a pellet and were incubated overnight (ON) with NPSR medium in U-bottom low-attachment 96-well plates (Thermo, Cat. No. 174929) at 37 °C and 5% CO_2_. After ON incubation, the cellular pellet was aggregated with freshly dissected and 30 mins hrGDNF (PeproTech) treated E11.5 UBs as described previously^36^. The aggregated pellets were centrifuged at 1000 rpm 4 min and kept in the 1.5 ml Eppendorf LoBind tubes with DMEM and 10% fetal bovine serum (FBS) medium ON. Next day, the pellets formed 3D aggregates at the bottom of the tubes and were transferred into a Trowell-type culture onto 0.1 μm or 1 μm pore polyester membrane and cultured for around 8 days in DMEM supplemented with 10% FBS medium at 37 °C and 5% CO_2_; medium was changed every other day.

### RT-PCR

An RNeasy kit (Qiagen) was used according to the manufacturer’s recommendations to extract the total RNA. cDNA synthesis (First Strand cDNA Synthesis Kit, ThermoFisher) was performed using standard protocols. qRT-PCR analyses were displayed with SYBR Green (Agilent) by an CFX96 Real-Time PCR machine. The Brilliant III SYBR® Green QPCR Master Mix (Agilent Technologies) was used according to the manufacturer’s instructions. The GAPDH probe served as a control to normalize the data. The gene expression experiments were performed in triplicates on three independent experiments. All the Primers sequences are given in Table S1.

### Whole mount immunostaining

For the immunostaining, the wild type and *Wnt4*^−*/*−^ kidney organoids were used at the same time, and treated as a control to each other. The kidney organoids were washed two times with 1x PBS and fixed with 100% cold Methanol (−20 °C pre-chilled) for 30 min, washed at least 3 times in 1x PBS before immunostaining. For immunostaining, the organoids were blocked in 0.1% Triton-X100, 1% BSA and 10% goat serum/0.02 M glycine-PBS for 1–3 hours at room temperature. Following blocking, the organoids were incubated ON in primary antibodies against Wt1 (1:100, #05–753, Millipore), Pax2 (1:200, #PRB-276P, Covance), in blocking buffer overnight at 4 °C. Next day, the organoids were washed with 1x PBS six times and incubated ON in 1x PBS with goat anti-rabbit IgG Alexa Fluor 546 (1:1000; #A11010, Life technologies), goat anti-mouse IgG Alexa Fluor 647 (1:1000; #A21235, Life technologies) and fluorescein anti-LTL (1:350, #FL-1321, Vector Laboratories) at 4 °C. A Zeiss LSM780 microscope and Zeiss Axiolab were used for image capture and analysis. Wild type and *Wnt4*^−*/*−^ kidney organoids were imaged using the same settings of the microscope.

## Electronic supplementary material

Supplementary Information

## Data Availability

The datasets generated and/or analyzed during the current study are available from the corresponding author on request.
