# Supplementary material for: Embryonic Stem Cells Derived Kidney Organoids as Faithful Models to Target Programmed Nephrogenesis
Source: Sci Rep. 2018 Nov 9;8:16618. doi: 10.1038/s41598-018-34995-3 (PMC6226521; doi:10.1038/s41598-018-34995-3)

Supplementary Information

Embryonic Stem Cells Derived Kidney Organoids as Faithful Models to Target Programmed Nephrogenesis

Zenglai Tan[1, *], Jingdong Shan[1], Aleksandra Rak-Raszewska[1] and Seppo J. Vainio[1*]

[1] Biocenter Oulu, Infotech Oulu, Center for Cell Matrix Research, Faculty of Biochemistry and Molecular Medicine, University of Oulu, Aapistie 5A, 90220 Oulu, Finland.

[*] Correspondence to:

Seppo Vainio, Email: seppo.vainio@oulu.fi; Zenglai Tan, Email: zenglai.tan@oulu.fi

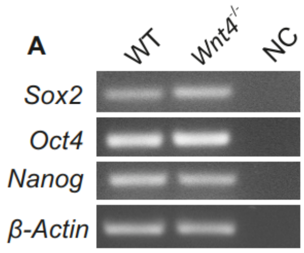

Supplementary Figure S1. RT-PCR shows WT and *Wnt4*$^{-/-}$ mESCs keep the stemness with the expression of stem cell markers: *Sox2, Oct4* and *Nanog*. NC: negative control with no DNA template.

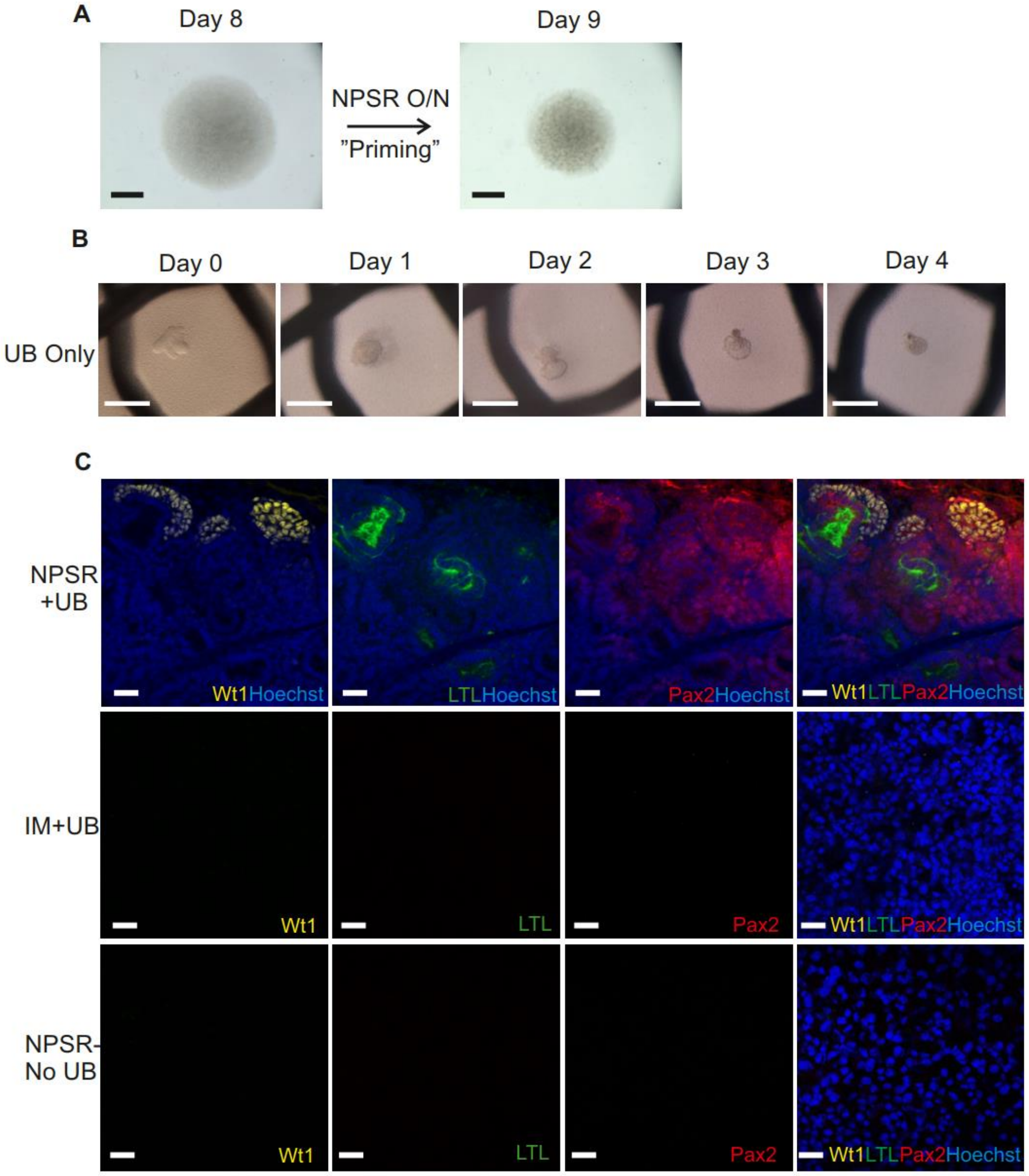

Supplementary Figure S2. Incubation of the IM cells in NPSR, trowell culture E11.5 ureteric bud (UB) and regulation of kidney organoid nephrogeneis. (A) Bright-field images showing the morphology of the cell pellet within NPSR medium overnight. Scale bars, 500 μm. (B) Time-lapse bright-field images of apoptosis of the E11.5 UB in Trowell culture. Scale bars, 500 μm. (C) Generation of kidney organoids using described protocol (upper panels). Omitted priming step, aggregates of the IM cells with UB for nephrogenesis didn't show nephron development (middle panels). The cell pellet after NPSR incubation and transfter to Trowell culture without aggregation with the UB display failure in kidney development (lower panels). Scale bars, 20 μm.

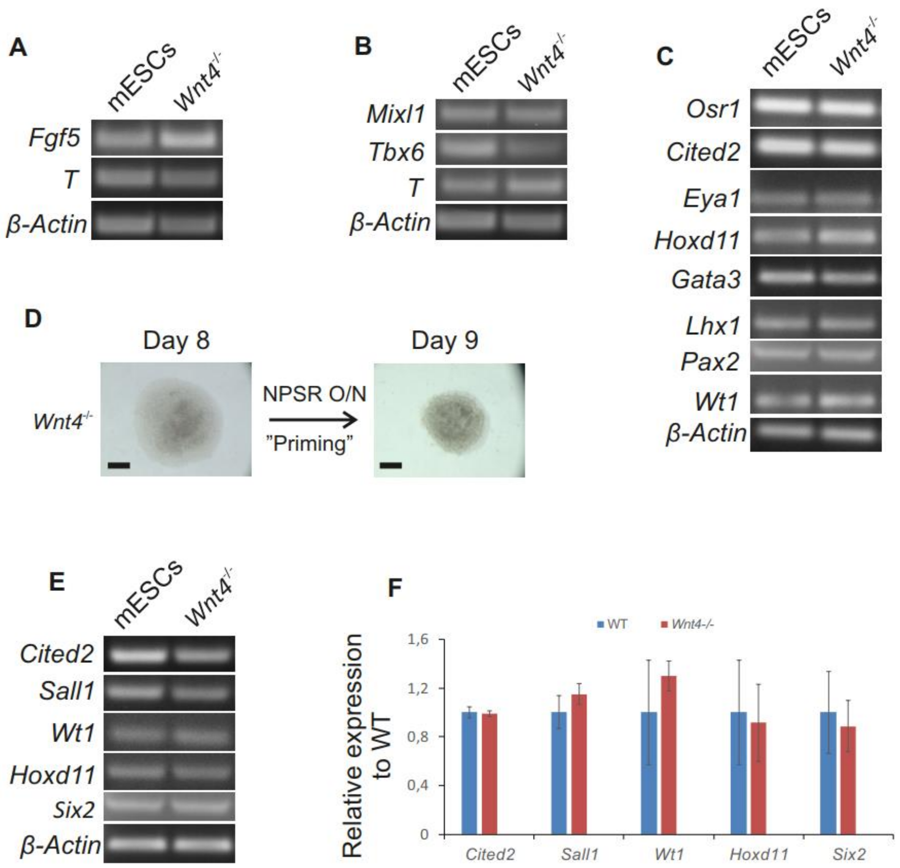

Supplementary Figure S3. Direct differentiation of *Wnt4*$^{-/-}$ mESCs to kidney lineage cells. (A) RT-PCR of wild type and *Wnt4*$^{-/-}$ mESCs differentiation presenting gene expression after 5 hours CHIR treatment; cells expressed epiblast markers *Fgf5* and *T*. (B) RT–PCR presenting gene expression during further CHIR differentiation of wild type and *Wnt4*$^{-/-}$ mESCs; day 2 showing the expression of primitive streak markers (*Mixl1*, *T*, *Tbx6*). (C) RT–PCR of wild type and *Wnt4*$^{-/-}$ mESCs at day 8 of differentiation showing the expression of markers of IM (*Osr1*, *Pax2*, *Lhx1*, *Gata3*, *Wt1*, *Eya1*, *Cited2*, *Hoxd11*). (D) Bright-field images showing the morphology of the *Wnt4*$^{-/-}$ cell pellets within NPSR medium overnight form. Scale bars, 500 µm. (E) RT–PCR of wild type and *Wnt4*$^{-/-}$ cell pellets expression of renal progenitor cell markers (*Cited2*, *Wt1*, *Sall1, Hoxd11*, *Six2*) after incubation overnight in NPSR medium. (F) qRT-PCR results show no signiciant different of wild type and *Wnt4*$^{-/-}$ cell pellets expression of renal progenitor cell markers (*Cited2*, *Wt1*, *Sall1, Hoxd11*, *Six2*) after incubation overnight in NPSR medium.

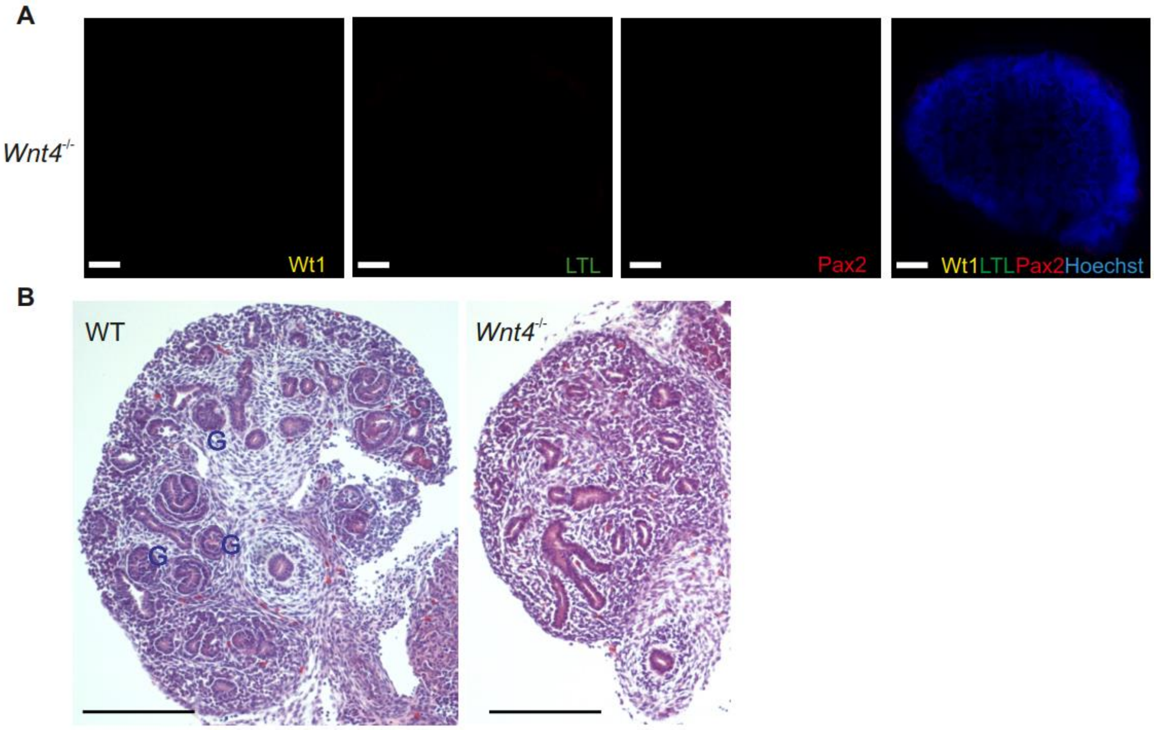

Supplementary Figure S4. Analysis of *Wnt4* mutant phenotypes. (A) whole-mount staining of the *Wnt4* knockout kidney organoids illustrating the failed kidney development, related to Fig 5C. Scale bars, 200 μm. (B) Hematoxylin and eosin staining shows the E14.5 wild-type *Wnt4* kidney (+/+) and *Wnt4* mutant kidney (-/-). G, glomerulus. Scale bars, 200 μm.

Supplementary Table S1. RT-PCR and qR-TPCR primers (5'-3')

Gene	Forward primer sequence	Reverse primer sequence
β-Actin	GATCTGGCACCACACCTTCT	GGGGTGTTGAAGGTCTCAAA
Fgf5	GCTGTGTCTCAGGGGATTGT	CACTCTCGGCCTGTCTTTTC
Afp	TCGTATTCCAACAGGAGG	AGGCTTTTGCTTCACCAG
T	GCTTCAAGGAGCTAACTAACGAG	CCAGCAAGAAAGAGTACATGGC
Mixl1	ACGCAGTGCTTTCCAAACC	CCCGCAAGTGGATGTCTGG
Tbx6	ATGTACCATCCACGAGAGTTGT	GGTAGCGGTAACCCTCTGTC
Pax6	TACCAGTGTCTACCAGCCAAT	TGCACGAGTATGAGGAGGTCT
Sox1	AAGGAACACCCGGATTACAAGT	GTTAGCCCAGCCGTTGACAT
Osr1	GAGCGACCTTACACCTGTGA	GTCTTGTGGACAGCGAGAGT
Lhx1	CAGTGTCGCCAAAGAGAACA	TGAGACGTTGGCACTTTCAG
Wt1	AAACCTGGAAACCTGGAAGG	GGCTCCTCTCCGTCCTAACT
Cited2	TGCAGAAGCTCAACAACCAG	CTGGTTTGTCCCGTTCATCT
Eya1	AATTTATGCCTGGCAACTGG	CAGACCTCCCACGTTGTTTT
Sall1	AGCCTCAACATTTCCAATCC	TGGGCATCCTTGCTCTTAGT
Gata3	CTCGGCCATTCGTACATGGAA	GGATACCTCTGCACCGTAGC
Hoxd11	TGGAACGCGAGTTTTTCTTT	TTGCAGACGGTCCCTGTTCA
Pax2	CGCCGTTTCTGTGACACACAATC	TGCTTGGGACCAAACACAAGGTG
Six2	AGGAAAGGGAGAACAGCGAGAA	GGACTGGACGACGAGTGGT
Oct4	CGGAAGAGAAAGCGAACTAGC	ATTGGCGATGTGAGTGATCTG
Nanog	TCTTCCTGGTCCCCACAGTTT	GCAAGAATAGTTCTCGGGATGAA
Sox2	CATCCACTTCTACCCCACCTT	AGCTCCCTGTCAGGTCCTT

Full gel images for Fig. 3C

NA (Not applicable)

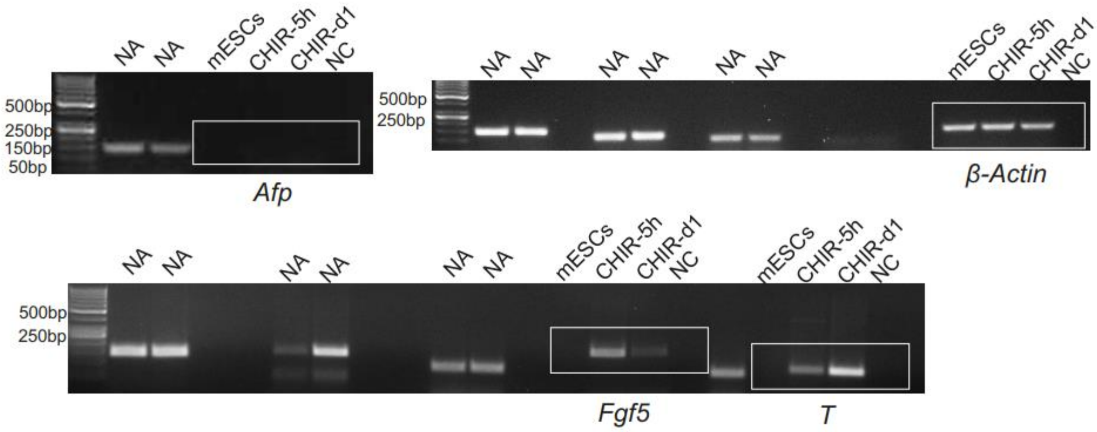

Full gel images for Fig. 3D

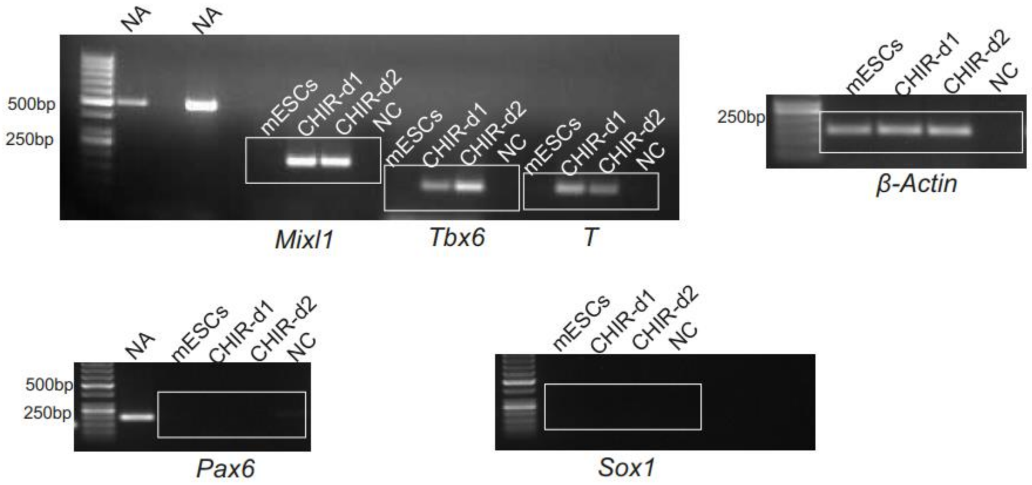

Full gel images for Fig. 3E

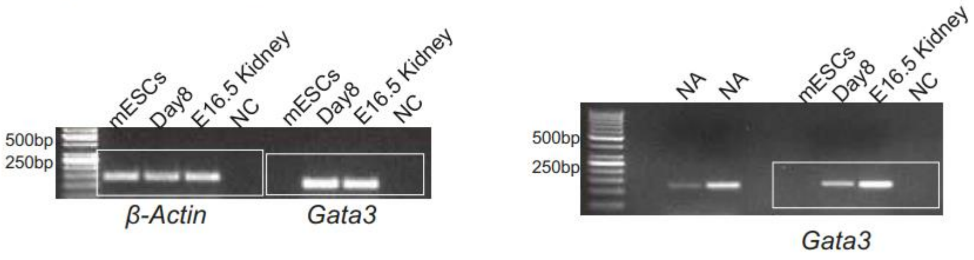

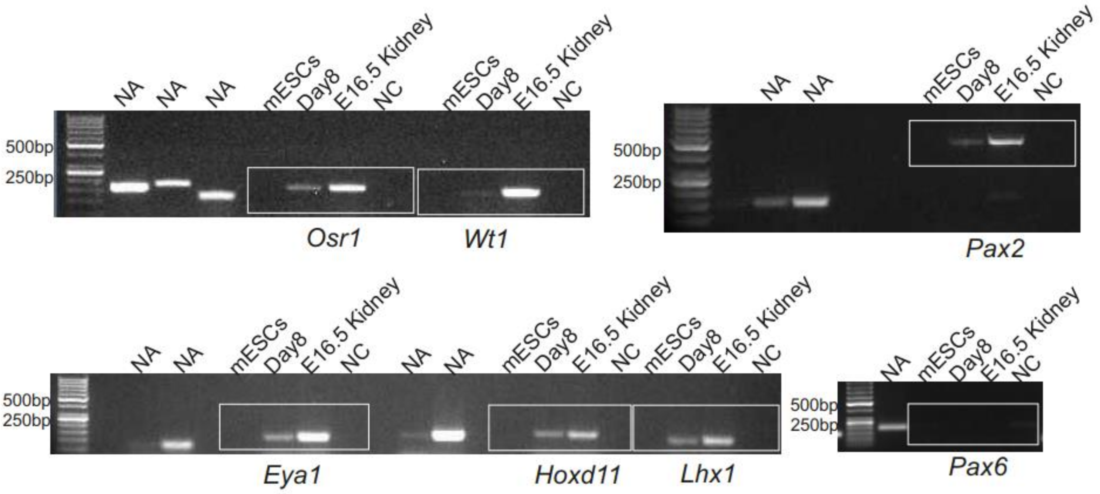

Full gel images for Fig. 4B

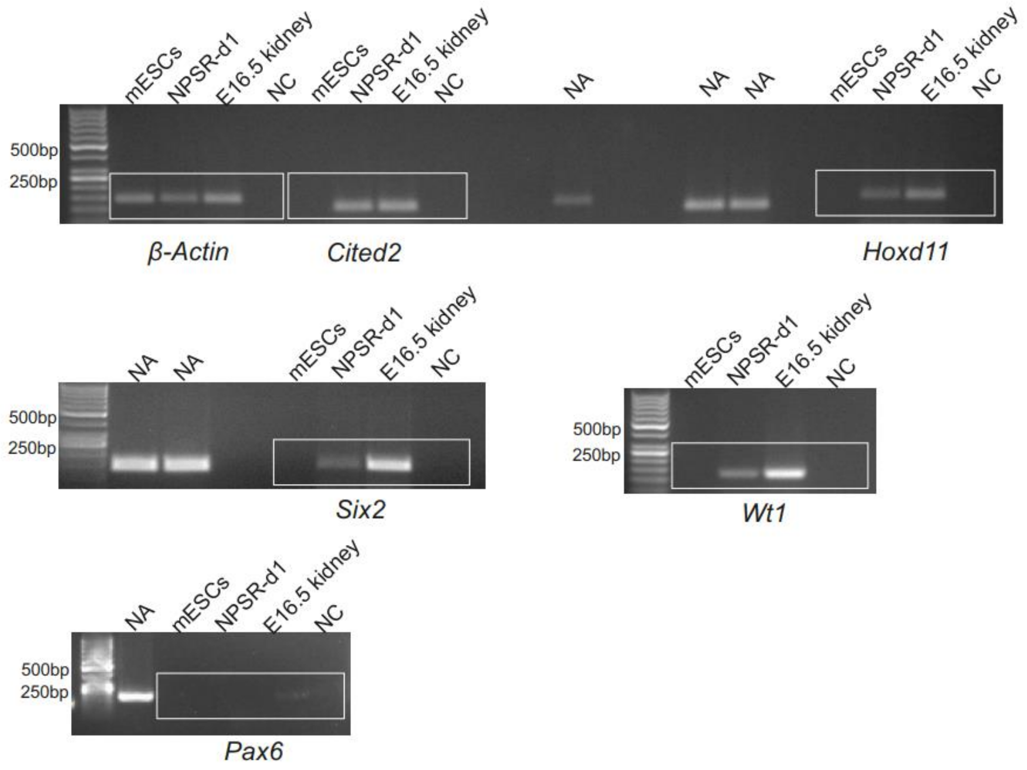

Supplement: Supplementary file 1 — Supplementary Information [file 41598_2018_34995_MOESM1_ESM.pdf]
